# Supplementary material for: Quantitatively assessing mekosuchine crocodile locomotion by geometric morphometric and finite element analysis of the forelimb
Source: PeerJ. 2020 Jun 15;8:e9349. doi: 10.7717/peerj.9349 (PMC7301899; doi:10.7717/peerj.9349)
Supplement: Supplemental Information 1 [file peerj-08-9349-s001.docx]

**Data S1**

**Description of Curves and Patches Used for Specimen Warping**

Primitives mapped to homologous landmarks used to warp specimens in the software Landmark IDAV version 3.0 (Wiley et al. 2005). Curves all consisted of 30 evenly-spaced (by length) landmarks.

(1) Curve 0: running along the proximal edge of the deltopectoral crest from the margin of the proximal epiphysis to the apex of the deltopectoral crest.

(2) Curve 1: running along the distal edge of the deltopectoral crest from the apex of the deltopectoral crest to surface of the diaphyseal shaft.

(3) Curve 2: running along the dorsolateral margin of the proximal head till level with termination of curve 1, center-point level with the apex of the deltopectoral crest.

(4) Curve 3: running along the ventromedial margin of the proximal head till level with termination of curve 1, center-point equidistant.

(5) Curve 4: running along the dorsomedial margin of the proximal head till level with termination of curve 1, center-point equidistant.

(6) Curve 5: running along the margin of the proximal epiphysis, dorsally on the proximal head, spanning the dorsal tuberosity.

(7) Curve 6: running along the margin of the proximal epiphysis, medially from curve 5 joining curve 4.

(8) Curve 7: running along the margin of the proximal epiphysis, laterally from curve 5 joining curve 2.

(9) Curve 8: running along the margin of the proximal epiphysis, laterally from curve 7 joining curve 0.

(10) Curve 9: running along the margin of the proximal epiphysis, medially from curve 6 joining curve 3.

(11) Curve 10: running along the margin of the proximal epiphysis between curves 8 and 9.

(12) Curve 11: running along the margin of the distal epiphysis, on the ventral surface spanning the curve of the lateral condyle from the lateral epicondyle to medial condyle.

(13) Curve 12: running along the margin of the distal epiphysis, medially from curve 11 to the medial epicondyle.

(14) Curve 13: running along the margin of the distal epiphysis, medially from curve 12 to the dorsal extend of the medial condyle.

(15) Curve 14: running along the margin of the distal epiphysis, lateral from curve 11 to the dorsal extend of the lateral condyle.

(16) Curve 15: running along the diaphyseal shaft from curve 1 to curve 11, center-point equidistant between these.

(17) Curve 16: running along the diaphyseal shaft from curve 4, terminating level with the lateral epicondyle, center-point level with the center-point of curve 15.

(18) Curve 17: running along the diaphyseal shaft from the midway between terminations of curves 1 and 3 to the center-point of curve 11, center-point level with the center-point of curve 15.

(19) Curve 18 running along the proximal margin of the proximal epiphysis between the center-point of curve 8 and the termination of curve 6.

(20) Curve 19: same as curve 11 but running level along the distal epiphysis.

(21) Curve 20: running along the distal surface of the lateral epicondyle from the center-point of curve 14 to the intercondylar sulcus, center-point situated on the apex of the lateral epicondyle.

(22) Curve 21: running along the distal surface of the medial epicondyle from curve 20 to the center-point of curve 13, center-point situated on the apex of the medial epicondyle.

(23) Patch 0: 5x5 landmarks, placed matching the shape of the small sulcus situated dorsally on the distal head between the condyles.

(24) Patch 1: 30x30 landmarks, placed between curves 0, 1, 3, 10 composing the ventral face of the proximal head.

(25) Patch 2: 30x30 landmarks, placed between curves 0, 1, 2, 8 composing the lateral face of the proximal head.

(26) Patch 3: 30x30 landmarks, placed between curves 2, 4, 5, 6, 7 composing the dorsal face of the proximal head.

**Output of Principal Component Analysis (PCA) Conducted on Geometric Morphometric Data (Body Mass Corrected Residuals) For Periosteal Contour Landmarks (LM) (N=16)**

**PCA Eigenvalues**

| PC | Eigenvalue | % variance |
| --- | --- | --- |
| 1 | 0.00164255 | 69.038 |
| 2 | 0.000492264 | 20.69 |
| 3 | 0.000168944 | 7.1009 |
| 4 | 2.89E-05 | 1.2136 |
| 5 | 2.86E-05 | 1.202 |
| 6 | 1.49E-05 | 0.62592 |
| 7 | 3.08E-06 | 0.1295 |
| 8 | 2.33E-13 | 9.81E-09 |

**PCA Scores**

| Specimen | PC1 | PC2 | PC3 | PC4 | PC5 | PC6 | PC7 | PC8 |
| --- | --- | --- | --- | --- | --- | --- | --- | --- |
| *C. porosus* XCb Cp4 | 1.48E-05 | -0.01905 | 0.000107 | 0.008476 | 0.005402 | -0.00348 | -0.00106 | 6.28E-07 |
| *C. johnstoni* AR 22025 | -0.092383 | 0.012959 | -0.01005 | 0.001051 | 0.003147 | 0.001422 | 0.000289 | -3.17E-07 |
| *C. johnstoni* AR 22161 | -0.023947 | -0.03252 | 0.021252 | 0.000287 | -0.00539 | 0.002375 | 0.001253 | -9.02E-08 |
| Murgon QM F56058 | 0.022394 | 0.019517 | 0.01575 | -0.00549 | 0.00883 | 0.002997 | 0.000161 | 2.06E-07 |
| Murgon QM F56060 | 0.011077 | 0.008049 | -0.00498 | -0.00352 | -0.00153 | -0.00667 | 0.003148 | 9.97E-08 |
| Riversleigh QM F57954 | 0.043136 | -0.00458 | -0.01903 | 0.002907 | -0.00141 | 0.006386 | 0.001392 | 1.22E-07 |
| Riversleigh QM F57955 | 0.030646 | 0.028944 | 0.008654 | 0.00698 | -0.00335 | -0.00129 | -0.00092 | -6.71E-07 |
| Floraville QM F57953 | 0.021372 | -0.02853 | -0.00906 | -0.00609 | 0.002191 | -0.00185 | -0.00197 | -6.39E-07 |
| Bullock Creek NTM P907-70 | -0.01231 | 0.01521 | -0.00264 | -0.0046 | -0.0079 | 0.000109 | -0.0023 | 6.62E-07 |

**PCA Loadings**

| Landmark (LM) | PC1 | PC2 | PC3 | PC4 | PC5 | PC6 | PC7 | PC8 |
| --- | --- | --- | --- | --- | --- | --- | --- | --- |
| LM1 | -0.01284 | -0.01403 | 0.03737 | -0.01005 | -0.1046 | 0.03708 | 0.06893 | -0.08977 |
| LM1 | -0.09722 | 0.2302 | -0.2074 | -0.0669 | -0.2744 | -0.08905 | -0.146 | -0.01676 |
| LM2 | -0.1111 | 0.03345 | -0.1304 | -0.06767 | -0.2316 | 0.1943 | 0.07813 | 0.09761 |
| LM2 | -0.2362 | 0.1172 | -0.3989 | -0.1261 | -0.2066 | 0.2578 | 0.1073 | 0.1028 |
| LM3 | -0.2345 | -0.07677 | -0.01275 | -0.1449 | 0.04483 | 0.1834 | -0.2147 | 0.3438 |
| LM3 | -0.2198 | -0.05767 | -0.04197 | -0.1196 | 0.2666 | 0.03166 | -0.2007 | -0.118 |
| LM4 | -0.2195 | -0.3354 | 0.3775 | 0.09864 | 0.2297 | 0.2277 | -0.262 | 0.1184 |
| LM4 | -0.08319 | -0.1314 | 0.147 | 0.06454 | 0.2452 | -0.03127 | -0.0142 | -0.1517 |
| LM5 | 0.1965 | -0.2043 | -0.0918 | 0.5147 | -0.07669 | -0.3632 | 0.1342 | 0.3821 |
| LM5 | 0.00257 | -0.00035 | 0.006635 | 0.009857 | 0.112 | -0.1143 | 0.1397 | 0.08226 |
| LM6 | 0.4094 | 0.01107 | -0.2949 | 0.06944 | 0.0454 | -0.09279 | -0.2336 | 0.03609 |
| LM6 | -0.172 | -0.00927 | 0.1448 | -0.01328 | 0.04822 | -0.05708 | 0.2593 | -0.09853 |
| LM7 | 0.2142 | 0.03343 | 0.0401 | -0.4935 | 0.2311 | -0.1747 | 0.2555 | 0.188 |
| LM7 | -0.2247 | -0.04833 | 0.005454 | 0.4954 | -0.2248 | 0.08645 | -0.01784 | -0.1222 |
| LM8 | 0.03864 | 0.07449 | 0.1126 | 0.04647 | -0.07101 | 0.06337 | 0.2176 | -0.07792 |
| LM8 | -0.1222 | -0.2196 | -0.1747 | -0.1221 | 0.02088 | -0.1987 | -0.1551 | 0.3862 |
| LM9 | -0.01247 | -0.01564 | 0.03748 | -0.00962 | -0.1016 | 0.03954 | 0.07975 | 0.1936 |
| LM9 | 0.2424 | -0.3515 | -0.03382 | -0.2269 | -0.3141 | 0.2318 | 0.1002 | -0.1905 |
| LM10 | 0.125 | -0.1059 | 0.08143 | -0.07356 | -0.1594 | 0.09145 | -0.08487 | -0.1649 |
| LM10 | 0.3354 | -0.2209 | 0.1122 | -0.1241 | -0.04469 | 0.03944 | -0.2864 | -0.01068 |
| LM11 | 0.1682 | 0.0849 | -0.04663 | 0.1218 | -0.0162 | 0.2015 | -0.1393 | -0.03524 |
| LM11 | 0.1824 | 0.1001 | -0.08169 | 0.1323 | 0.2024 | 0.02872 | -0.07493 | -0.3611 |
| LM12 | 0.05852 | 0.45 | -0.1423 | 0.1131 | 0.325 | 0.2911 | -0.06228 | 0.118 |
| LM12 | 0.03212 | 0.1954 | -0.06606 | 0.06914 | 0.287 | -0.01426 | 0.07059 | 0.02257 |
| LM13 | -0.1512 | 0.3583 | 0.3648 | -0.06381 | -0.2102 | -0.2192 | -0.2251 | 0.05809 |
| LM13 | 0.002337 | 0.000954 | 0.00685 | 0.01193 | 0.1129 | -0.1173 | 0.1396 | -0.1949 |
| LM14 | -0.2373 | -0.01296 | 0.01984 | -0.09551 | -0.02947 | -0.3317 | 0.224 | -0.1379 |
| LM14 | 0.09604 | -0.00049 | 0.01456 | 0.0519 | 0.07904 | 0.04116 | 0.07489 | 0.1739 |
| LM15 | -0.1838 | -0.1608 | -0.304 | -0.0395 | 0.08931 | -0.2684 | -0.08772 | -0.2625 |
| LM15 | 0.1723 | 0.1479 | 0.3455 | 0.04532 | -0.07592 | 0.181 | 0.2917 | 0.1598 |
| LM16 | -0.04783 | -0.1199 | -0.04829 | 0.03407 | 0.03548 | 0.1204 | 0.2519 | 0.008643 |
| LM16 | 0.08971 | 0.2479 | 0.2215 | -0.08144 | -0.2336 | -0.276 | -0.2883 | -0.03003 |

**References**

Wiley DF, Amenta N, Alcantara DA, Ghosh D, Kil YJ, Delson E, Harcourt-Smith W, Rohlf FJ, St John K, Hamann B. 2005. Evolutionary morphing. *Visualization* 5, 431–438. DOI: 10.1109/VISUAL.2005.1532826. Program available at http://graphics.idav.ucdavis.edu/research/ EvoMorph.
